# Overweight, obesity, physical activity and sugar-sweetened beverage consumption in adolescents of Pacific islands: results from the Global School-Based Student Health Survey and the Youth Risk Behavior Surveillance System

**DOI:** 10.1186/s40608-015-0062-4

**Published:** 2015-09-16

**Authors:** Tara Kessaram, Jeanie McKenzie, Natalie Girin, Onofre Edwin A. Merilles, Jessica Pullar, Adam Roth, Paul White, Damian Hoy

**Affiliations:** Public Health Division, Secretariat of the Pacific Community, BP D5 98848 Noumea, New Caledonia

## Abstract

**Background:**

Overweight, obesity and their consequences are challenges to sustainable social and economic development in Pacific island countries and territories (PICTs). Complementing previous analyses for adults, the purpose of this paper is to synthesise available data on overweight, obesity and their risk factors in adolescents in the region. The resulting Pacific perspective for the younger generation will inform both the national and regional public health response to the crisis of noncommunicable diseases.

**Methods:**

We examined the prevalence of overweight, obesity, physical activity and carbonated sugar-sweetened beverage (SSB) consumption, by using published results of two cross-sectional surveys: the Global School-Based Student Health Survey (GSHS) and the Youth Risk Behavior Surveillance System (YRBSS). GSHS was conducted in ten PICTs between 2010 and 2013 and provided results for 13–15 year olds. YRBSS surveys, conducted repeatedly in five PICTs between 1999 and 2013, provided results for grade 9–12 students (approximately 14–18 years) and enabled examination of trends.

**Results:**

Obesity prevalence ranged from 0 % in female students in Vanuatu to 40 % in males in Niue (GSHS). Among grade 9–12 students (YRBSS), obesity was highest in American Samoa (40 % of males; 37 % of females). Approximately 60 % of students in the Cook Islands, Niue and Tonga (GSHS) and American Samoa (YRBSS), were overweight. In both surveys, less than half of students reported engaging in sixty minutes of physical activity on at least 5 days of the past week. Daily consumption of carbonated SSBs in the past month was reported by over 42 % of students in six PICTs (GSHS), and in the past week by more than 18 % of students in three PICTs (YRBSS). In PICTs conducting YRBSS, obesity prevalence remained high or increased within the period 1999–2013.

**Conclusion:**

There is a need for urgent action on overweight, obesity and their risk factors in Pacific youth. The multiple social, economic and physical determinants of this public health crisis must be addressed. This requires all sectors within government and society in PICTs to implement and evaluate policies that will protect and promote the health of their populations across the life course.

## Background

For over 60 years, 22 Pacific island countries and territories (PICTs), referred to here as the Pacific region, have worked together on issues of health and development, as members of the Secretariat of the Pacific Community [1]. In 1995, through the Yanuca Island Declaration, Ministers of Health representing these PICTs set forth the vision of Healthy Islands, a “unifying theme for health protection and health promotion in the island nations of the Pacific for the twenty-first century [2].” Twenty years later, PICTs have declared a crisis of noncommunicable diseases (NCD) [3]. Risk factors for NCDs, including high body mass index (BMI), are leading contributors to death and disability in the region [4].

Globalisation, trade liberalisation and increasing urbanisation are contributing to nutrition and physical activity transitions [5–7]. In the Pacific, traditional diets of root crops, vegetables, fruits, fresh fish and meat are being replaced by imported and processed energy dense, low nutrient foods [8–11]. The availability, affordability and convenience of these products promotes their purchase and consumption [12, 13]. Increasing demand and reliance on processed foods may also threaten local farming and fishing practices and the extensive opportunities for physical activity that these occupations provide. Further, urban living in the islands can be associated with lower levels of work and travel related physical activity [14].

The impact of these changing environments on younger generations is concerning. Obesity in youth adversely affects their psychological, musculoskeletal, cardiovascular and respiratory health [15, 16]. Furthermore, it is associated in the long-term with adult obesity, Type II diabetes, hypertension, cardiovascular morbidity and premature mortality [17].

Adult overweight and obesity prevalence in PICTs has recently been described [18]. To complement this, the objective of this paper is to synthesise, present and interpret available data for adolescents from two school-based surveys of obesity, overweight, physical activity and consumption of carbonated sugar-sweetened beverages (SSBs), across 15 PICTs. This work will inform national and regional NCD strategies and action plans, assist with evaluation of their impact and help track progress in the Pacific towards the global target of halting the rise in obesity [19].

## Methods

### Setting

The Melanesian, Polynesian and Micronesian islands of the Pacific region are culturally and socioeconomically diverse. The populations of PICTs vary in size; Tokelau has a population of 1400, while there are over 7 million in Papua New Guinea (PNG). In Kiribati, Nauru and PNG, life expectancy at birth is under 65 years, whereas it exceeds 78 years in Guam [20]. Enrolment in secondary school ranges across the region; for example, the net enrolment ratio is 13 % in PNG and 89 % in Palau [20].

### Data sources and study design

We selected the World Health Organization (WHO) Global School-Based Student Health Survey (GSHS) [21] and the Centers for Disease Control and Prevention (CDC) Youth Risk Behavior Surveillance System (YRBSS) [22] as the data sources for this work. Participating countries and survey years are listed in Table 1. GSHS has been conducted in ten PICTs, and YRBSS in five PICTs which are also known as United States affiliated Pacific Islands (USAPIs). These surveys provided the widest coverage of the region and the opportunity for two subregional analyses on account of the standardised methodologies used.

**Table 1:** GSHS and YRBSS surveys undertaken in the Pacific and included in this analysis

	Global School Based-Student Health Survey (GSHS)	Youth Risk Behavior Surveillance System (YRBSS)
American Samoa		1999 2007 2011
Commonwealth of the Northern Mariana Islands		2003 2005 2007
Cook Islands	2011	
Fiji	2010	
Guam		2001 2007 2011 2013
Kiribati	2011	
Nauru	2011	
Niue	2010	
Palau		1999 2001 2003 2005 2007 2009 2011
Republic of the Marshall Islands		2003 2007
Samoa	2011	
Solomon Islands	2011	
Tonga	2010	
Tuvalu	2013	
Vanuatu	2011	

#### Global School-Based Student Health Survey

Of the ten PICTs conducting GSHS between 2010 and 2013, seven employed a two-stage sampling design to obtain a representative sample of students. Schools were selected by probability proportional to size and then classes within these schools were selected randomly. All students in these classes were eligible to participate. In the Cook Islands, Nauru and Niue, a census was used to obtain data on the target student population.

Local policies and practices govern whether parental approval is required for students to participate in GSHS. Parents are usually notified of the survey in writing and permission requested as needed [23]. Students aged 13–17 years in the selected classes complete a self-administered, anonymous questionnaire which includes questions on demographics, diet and physical activity. Survey staff measure participants’ height and weight beforehand and provide these results in writing to each individual for them to transfer to their computer-scannable answer sheet. Data are weighted to account for sampling design, population distribution by grade and sex, and non-response. A response rate of over 60 % is required for results to be weighted and therefore generalized to the target population [24]. The surveys included in this analysis had overall response rates of 72 % or higher. We extracted data from factsheets of GSHS which provided results for the subset of the survey population aged 13–15 years [25–34].

#### Youth Risk Behavior Surveillance System

YRBSS surveys have been conducted at least twice in five USAPIs between 1999 and 2013. Local polilcies guide procedures for gaining parental permission and may include obtaining written consent from parents for students to take part in the survey [35]. The sampling design and analysis of YRBSS are similar to GSHS [24, 35]. Generally, in USAPIs apart from Guam, surveys were designed to include all members of the target population. In YRBSS, students in grades 9–12 (approximately 14–18 years) complete an anonymous questionnaire on a range of risk and protective behaviours. Height and weight are self-reported. A response rate of 60 % or more is required for results to be weighted [35]. We extracted results primarily from CDC’s database, Youth Online [22]. For CNMI, we also used results published by Lippe et al. [36]. For each USAPI, we examined the most recent year for which sex-specific weighted data were available. We also analysed trends over time in overweight and obesity prevalence.

### Variables

The variables used for this analysis and their definitions are listed in Table 2. Overweight and obesity (defined by WHO as abnormal or excessive fat accumulation that presents a risk to health) [37] are classified in GSHS and YRBSS according to Body Mass Index (BMI)-for-age-and-sex WHO 2007 and CDC 2000 growth references, respectively [38, 39]. In GSHS, overweight is defined as > +1 standard deviation from the median BMI and obesity as > +2 standard deviations. In YRBSS, overweight is reported as a BMI ≥85th and <95th percentile, and obesity as a BMI ≥95th percentile. For the first part of our analysis, we added the prevalence of overweight and obesity from YRBSS to match the GSHS concept of overweight. For the trend analysis of YRBSS data, we examined overweight and obesity according to their standard definitions. We selected time spent in physical activity on account of the well-established evidence base for association with overweight and obesity, the global recommendation (60 mins of moderate to vigorous intensity activity daily for adolescents) and the global voluntary target (to reduce the prevalence of insufficiently physically active adolescents by 10 % by 2025) [19, 40, 41]. Consumption of sugar-sweetened beverages (SSBs), which include soda/soft drinks, juices and flavoured milks, is also an important risk factor for overweight and obesity and related diseases such as type 2 diabetes [42]. The World Health Organization has recently strongly recommended free sugars intake be reduced to less than 10 % of total energy intake [43]. Both GSHS and YRBSS examined daily carbonated SSB consumption (sodas/soft drinks, excluding diet versions) over the past month and week, respectively.

**Table 2:** Selected indicators from GSHS and YRBSS

	Global School-Based Student Health Survey	Youth Risk Behavior Surveillance System
Overweight^a^	Percentage of students who were > +1 SD from median for BMI by age and sex.	Percentage of students who were ≥85th and <95th percentile for BMI by age and sex.
Obesity^a^	Percentage of students who were > +2 SD from median for BMI by age and sex.	Percentage of students who were ≥95th percentile for BMI by age and sex.
Physical activity^b^	Percentage of students who were physically active for a total of at least 60 min per day on five or more days during the past seven days.	Percentage of students who were physically active at least 60 min per day on five or more days during the seven days before the survey.
Sugar-sweetened beverage consumption^c^	Percentage of students who usually drank carbonated soft drinks one or more times per day during the past 30 days.	Percentage of students who drank a can, bottle, or glass of soda or pop one or more times per day during the seven days before the survey.

*SD* standard deviation, *BMI* body mass index

^a^Overweight and obesity in GSHS and YRBSS are defined using World Health Organization (2007) and Centers for Disease Control (CDC) (2000) growth reference charts, respectively. Height and weight were self-reported in YRBSS

^b^GSHS questionnaires for Fiji and Nauru advised students to exclude time spent in physical education/gym class. GSHS and YRBSS define physical activity in the questionnaires as “any activity that increases your heart rate and makes you get out of breath some of the time” and provided examples

^c^GSHS and YRBSS questionnaires provided examples of carbonated soft drinks and asked students not to include diet soft drinks/soda/pop

Ethical approval for this work was not sought as data used were aggregate, unidentifiable and accessible within the public domain.

## Results

### Overweight and obesity

Among students aged 13–15 years (GSHS), prevalence of overweight exceeded 50 % in males in three PICTs, and in females in four PICTs (see Table 3). For males, prevalence was highest in Tonga, Niue, the Cook Islands and Samoa, and for females in Samoa, the Cook Islands, Tonga and Tuvalu. In contrast, the percentage of overweight students was below 23 % for both sexes, in Fiji, the Solomon Islands and Vanuatu. In the USAPIs for those in grades 9–12 (YRBSS), overweight prevalence was highest in American Samoa (61 % of males, 60 % of females), and lowest in Palau where approximately a quarter of students were overweight (Table 3). Gender differences observed by YRBSS were less marked than in GSHS in which 13–15 year old females had a higher point prevalence of overweight in eight PICTs.

**Table 3:** Overweight and obesity^a^ prevalence in the Pacific Region, GSHS and YRBSS, 2007-2013

Global School-Based Student Health Surveys (GSHS) (13-15 years)				
	Overweight (including obese)		Obese	
Country	Males	Females	Males	Females
	% (95 % CI)	% (95 % CI)	% (95 % CI)	% (95 % CI)
Cook Islands 2011	58.2	58.9	29.0	19.0
Fiji 2010	17.9 (13.0–24.2)	20.4 (16.1–25.4)	5.9 (4.0–8.8)	4.5 (3.0–6.7)
Kiribati 2011	31.9 (27.1–37.1)	46.4 (41.9–51.0)	7.8 (5.0–12.0)	8.5 (6.8–10.5)
Nauru 2011	40.0	48.9	17.8	15.7
Niue 2010	60.3	---	39.9	---
Samoa 2011	43.4 (39.0–47.9)	59.1 (55.5–62.6)	15.7 (12.4–19.6)	22.3 (19.1–25.8)
Solomon Islands 2011	17.6 (13.1–23.2)	22.4 (16.7–29.5)	1.5 (0.7–3.1)	2.9 (1.2–6.7)
Tonga 2010	61.2 (57.7–64.6)	58.0 (53.8–62.0)	24.7 (21.9–27.7)	19.1 (15.8–22.8)
Tuvalu 2013	44.3	52.2	22.1	20.9
Vanuatu 2011	8.9 (4.3–17.5)	13.6 (8.0–22.0)	0.3 (0.0–2.2)	0.0
Youth Risk Behavior Surveillance System (YRBSS) (Grades 9–12)				
	Overweight (including obese)		Obese	
Country	Males	Females	Males	Females
	% (95 % CI)	% (95 % CI)	% (95 % CI)	% (95 % CI)
American Samoa 2011	60.7	59.8	40.4	37.3
Guam 2013	37.2	36.8	22.8 (18.9–27.2)	17.1 (14.1–20.6)
Palau 2011	24.4	24.8	12.5	11.5
Commonwealth of the Northern Mariana Islands 2007	32.3	30.4	16.0	12.9
Republic of the Marshall Islands 2007	38.7	40.4	26.3	23.4

Confidence intervals were not reported for Cook Islands, Nauru, Niue, American Samoa, Republic of the Marshall Islands, Commonwealth of the Northern Mariana Islands and Palau, as surveys were designed to include all members of the target population. Confidence intervals were not reported for Tuvalu

Prevalence for females in Niue was not reported due to small sample size (less than 20 students)

Results for CNMI for 2007 were obtained from: Lippe J, Brener N, Kann L. et al. Youth risk behavior surveillance - Pacific Island United States Territories, 2007 MMWR Surveillance Summaries. 2008;57(12):28–56

*CI* Confidence Interval

^a^Overweight was defined in GSHS as Body Mass Index (BMI) > +1 standard deviation from the median for BMI for age and sex based on World Health Organization reference. Obesity was defined in GSHS as BMI > +2 standard deviations from the median. For the United States affiliated Pacific Islands, we estimated overweight from the addition of YRBSS results for overweight (BMI ≥85th and <95th for BMI for age and sex based on the 2000 Centers for Disease Control and Prevention reference) and obesity (defined as ≥95th percentile for BMI for age and sex). Height and weight were self-reported in YRBSS

Among males aged 13–15 years, obesity was virtually absent in Vanuatu, but affected 40 % in Niue. In six PICTs, over 15 % of males in this age range were obese. Prevalence of obesity in females was highest in Samoa, affecting 22 % (95 % CI: 19–26) of students, and exceeded 15 % in five PICTs. Differences between the sexes varied among PICTs; in three islands, the point prevalence of obesity was higher in females. Among those in grades 9–12 in the USAPIs, obesity prevalence was highest in American Samoa (40 % of males, 37 % of females). Prevalence was lowest in females in Palau (12 %). Overall, more males than females were obese in the USAPIs (Table 3).

### Trends (YRBSS, grade 9–12 students)

**Fig. 1:**
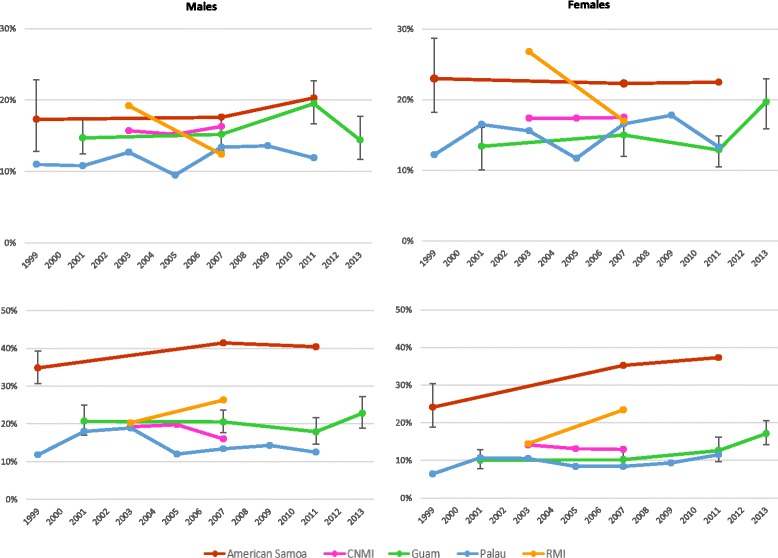
Prevalence of overweight (above) and obesity (below) in grade 9 to 12 students, 1999 to 2013. Source: Youth Risk Behavior Surveillance System (YRBSS) in United States affiliated Pacific Islands between 1999 and 2013. CNMI: Commonwealth of the Northern Mariana Islands. RMI: Republic of the Marshall Islands. Overweight was defined as ≥85th and < the 95th percentile for body mass index (BMI) by age and sex. Obesity was defined as ≥95th percentile for BMI for age and sex. Height and weight were self-reported. Confidence intervals were reported for Guam and American Samoa (1999) only as other surveys in American Samoa, CNMI, Palau and RMI were designed to include all members of the target student population

There was a statistically significant increase in the prevalence of overweight in females in Guam between 2001 and 2013 (Fig. 1). In American Samoa, CNMI and Palau, prevalence of overweight was consistently high at 9.5 % or more within the period of study. For both sexes in RMI, there was a steep decline in overweight prevalence and a marked increase in obesity prevalence, between 2003 and 2007. There were also statistically significant increases in obesity prevalence among females in Guam between 2001 and 2011, and in American Samoan males and females between 1999 and 2011.

### Physical activity

Less than half of students aged 13–15 years in nine PICTs engaged in sixty minutes of physical activity on five or more days of the past week (Table 4). Prevalence of physical activity ranged widely between islands. For both sexes, around 45 % of students were physically active in Vanuatu. In contrast, 18 % of males and 13 % of females were physically active in Nauru. Among grade 9–12 students in the USAPIs, prevalence of physical activity was highest in males in Guam (44 % 95 % CI: 40–48). Over a third of students were physically active in Palau, and over 20 % were physically active in American Samoa. Males were more active than females in the USAPIs, as observed in other PICTs by GSHS.

**Table 4:** Physical activity^a^ and daily soda/soft drink consumption^b^ prevalence in the Pacific Region, GSHS and YRBSS, 2010–2013

Global School-Based Student Health Survey (GSHS)				
	Physical activity^a^		Soft drink consumption^b^	
	Males	Females	Males	Females
	(95 % CI)	(95 % CI)	(95 % CI)	(95 % CI)
Cook Islands 2011	44.3	34.5	59.7	63.6
Fiji 2010	37.1 (31.8–42.8)	30.0 (24.2–36.6)		
Kiribati 2011	32.8 (27.7–38.3)	21.9 (18.8–25.3)	18.4 (14.0–23.7)	25.6 (22.7–28.7)
Nauru 2011	17.5	12.5		
Niue 2010	41.4		73.9	
Samoa 2011	19.8 (17.1–22.8)	22.1 (19.2–25.2)	54.6 (50.1–59.0)	52.5 (48.6–56.4)
Solomon Islands 2011	30.0 (25.4–35.0)	25.1 (18.0–33.8)	46.4 (39.1–53.7)	42.5 (35.6–49.6)
Tonga 2010	25.6 (21.0–30.9)	25.7 (22.7–29.0)	56.2 (52.0–60.3)	57.8 (54.3–61.1)
Tuvalu 2013			48.5	55.0
Vanuatu 2011	45.7 (32.1–60.0)	46.7 (30.0–64.1)	37.2 (27.1–48.5)	38.5 (30.9–46.8)
Youth Risk Behavior Surveillance System (YRBSS)				
	Physical activity^a^		Soft drink consumption^b^	
	Males	Females	Males	Females
	(95 % CI)	(95 % CI)	(95 % CI)	(95 % CI)
American Samoa 2011	27.5	21.7	30.3	38.7
Guam 2013	44.0 (39.7–48.3)	30.2 (24.7–36.3)	18.7 (15.3–22.6)	19.2 (15.5–23.4)
Marshall Islands 2007				
Commonwealth of the Northern Mariana Islands 2007				
Palau 2011	37.6	35.7	23.7	28.9

Confidence intervals were not applicable to results from the Cook Islands, Nauru, Niue, American Samoa, Commonwealth of the Northern Mariana Islands (CNMI), Republic of the Marshall Islands (RMI) and Palau, as surveys were designed to include all members of the target population. Confidence intervals were not reported for Tuvalu results

Results for females in Niue were not reported on account of small sample size (less than 20 students)

Physical activity results were not available for Tuvalu, RMI and CNMI. Soft drink consumption results were not available for Fiji, Nauru, RMI and CNMI

Results for CNMI for 2007 were obtained from: Lippe J, Brener N, Kann L. et al. Youth risk behavior surveillance - Pacific Island United States Territories, 2007 MMWR Surveillance Summaries. 2008;57(12):28–56

*CI* confidence interval

^a^In GSHS and YRBSS, this indicator was the proportion of students who were physically active (defined as engaging in any activity that increases the heart rate and makes one get out of breath some of the time) for at least 60 min per day on at least five of more days during the past seven days. Questionnaires for Fiji and Nauru advised students to exclude time spent in physical education/gym class

^b^GSHS reported the percentage of students who drank carbonated soft drinks one or more times per day during the past 30 days. YRBSS reported the percentage of students who drank a can, bottle or glass of soda or pop one or more times per day during the 7 days before the survey

### Daily carbonated sugar-sweetened beverage consumption (soft drink/soda)

In seven of the eight PICTs for which data were available, over 37 % of 13–15 year old students consumed at least one carbonated soft drink/soda per day over the course of the past 30 days (Table 4). Prevalence of daily consumption was lowest in Kiribati and highest in Niue where 74 % of males drank soft drinks every day. Prevalence was often similar between the sexes, though notably higher in females in the Cook Islands (64 %) and Tuvalu (55 %). The proportion of students in grades 9–12 who drank soda at least once per day in the past week varied in the three USAPIs for which data were available. Prevalence ranged from a low of 19 % (95 % CI: 15–23) of males in Guam, to 39 % of female students in American Samoa. Notably more females than males in Palau and American Samoa drank soda daily.

## Discussion

Overweight and obesity are determined by individual factors and risk behaviours, which are in turn affected by the interaction of economic and political forces with local environments [44]. The high prevalence of overweight and obesity in Pacific adolescents demonstrates the gravity of the public health problem in the region. Further, prevalences of overweight and obesity in USAPIs have remained high over time, with increases observed in the latter. Less than half of youth obtain sixty minutes of physical activity on five days of the past week, indicating many are not meeting the global recommendation [41]. In addition, there is excessive consumption of carbonated SSBs among students across the region.

### Regional and international comparisons

Estimates for the Global Burden of Disease (GBD) Study 2013 indicate that the prevalence of overweight and obesity in youth (under 20 years) in developed countries has risen between 1980 and 2013, from 16.9 to 23.8 % in males, and 16.2 to 22.6 % of females. In developing countries prevalence has risen from 8.1 to 12.9 % in males, and from 8.4 to 13.4 % in females [45]. Some of the highest estimates of obesity in the GBD study for this age group were in Pacific islands - 22.9 % of males and 36.0 % of females in Kiribati, and 23.7 % of males and 29.6 % of females in Samoa [45]. This finding is reflected in the results presented here, and echoed in national surveys undertaken in other PICTs. In a 2010 cross-sectional survey of the oral health of school children in New Caledonia, 20.5 % of 12 year olds were obese (WHO growth reference) [46]. In French Polynesia, the national prevalence of obesity (WHO growth reference) in 2008 was 21.4 % in boys and 19.2 % in girls 5–14 years [47].

Regarding risk factors for overweight and obesity, GSHS results across 34 countries revealed that only 23.8 % of boys and 15.4 % of girls were physically active for 60 mins on at least five days of the week [48]. From the present analysis, in comparison more students were physically active in the PICTs conducting GSHS. Fewer male students in the USAPIs were physically active compared to the 2013 YRBSS national United States estimate (57.3 %) [22]. The prevalence of daily consumption of carbonated soft drinks (not including diet soda) in students aged 13–15 years in PICTs was akin to that in the Eastern Mediterranean WHO Region where it ranged from 30.8 % in the Syrian Arab Republic to 74.3 % in Kuwait [49].

### Strengths and limitations

By collating the most recent available and comparable data from GSHS and YRBSS across 15 PICTs, this work fills a critical gap in evidence on overweight, obesity and their associated risk factors in Pacific adolescents. The analysis presented is based on the information provided from the data sources, in addition to resource guides on the general methodology of these surveys.

Although this work provides an important regional perspective on adolescent obesity, our analysis is limited due to incomplete coverage of the Pacific; critically, it does not include representative data for the most populous nation, Papua New Guinea. In addition, the prevalence of obesity and associated risk factors may have changed since the surveys used for our analysis were undertaken. Of note, more recent sex-specific YRBSS results from CNMI, RMI, Palau and American Samoa were not available through Youth Online.

Both GSHS and YRBSS are subject to potential biases. GSHS and YRBSS are representative only of those in formal education and at school on the day of the survey. As risk factor behaviour is self-reported, the surveys may underestimate or overestimate prevalence according to the social desirability of response. Further, it is known that YRBSS can underestimate the prevalence of obesity due to participants under-reporting their weight and over-reporting their height [50]. Regarding reliability, in a small sample of indigenous Fijian adolescent females, the test-retest reliability for GSHS questions on dietary behaviour was found to be lower than that for other risk behaviour domains, suggesting caution may be needed in interpretation of GSHS results [51].

Due to the different growth references from WHO and CDC used in the Pacific, we are only able to examine overweight and obesity prevalence at the sub-regional level. Estimates of the population prevalence of overweight and obesity are affected by the reference used; the WHO 2007 growth reference can yield higher estimates compared to the CDC 2000 growth reference [52]. Therefore, applying the WHO reference to the USAPIs may produce higher overweight and obesity estimates than those observed currently. This hypothesis and the effect of different references on Pacific data warrant further formal investigation.

We have not examined inequalities by ethnicity, socioeconomic status or rural/urban residence in this analysis, and have only studied a limited number of risk factors. Analyses of disparities and examination of a wider range of risk and protective behaviours would enable a more comprehensive understanding of overweight and obesity in Pacific youth. Importantly, further statistical analysis is essential to understand the magnitude of the contribution of all risk factors to the prevalence of overweight and obesity observed, in order to shape policy. Lastly, this work is focused on adolescents; national prevalence data on overweight and obesity in early childhood is required to inform the life course approach to prevention.

### Implications for policy and practice

The evidence presented justifies concern for the present and future health of Pacific youth and warrants decisive preventative action on obesity. Such action must fundamentally address the global and national drivers outlined earlier, as well as the more proximal food, physical and socio-cultural contexts which affect diet and exercise patterns of youth in PICTs. For example, in Tonga and Fiji, the availability and ease of access to food and drinks high in fat and sugar near schools and within their canteens enables youth to purchase unhealthy options [53, 54]. With regards to physical activity, safety concerns, poorly maintained roads, and the distance to recreational facilities are frequently cited barriers to engaging in exercise by youth in Apia, Samoa [55]. Culture and social values, such as the centrality of food to express nurture and care, higher esteem afforded to imported foods, and structures of rank and status may differentially influence the type and amount of food consumed within communities in the Pacific [12, 56, 57]. For indigenous Fijian and Tongan males, desire and pressure to perform well in sport and to achieve a robust muscular body may affect eating and exercise accordingly [57]. In contrast, females’ opportunities for physical activity may be constrained by gendered social norms [56].

With cognisance of this context, change at the national level, as well as across multiple-settings in communities, is essential. To increase the availability and affordability of healthier food and drink options in communities, PICTs can consider investing in local agriculture and fisheries, constructing tariff schedules to incentivise the importation and consumption of healthier products, and restricting imports of unhealthy foods [7]. Precedent in the Pacific exists - several PICTs have imposed taxes on SSBs for health benefits and/or revenue raising potential [58]. Tokelau has banned the importation of soft drinks [59]. These policy interventions may contribute to reductions in free sugars intake in accordance with WHO recommendations. Domestic policy space may, however, be constrained by international trade agreements, as demonstrated by the reversal of Samoa’s ban on the importation of turkey tails in order to comply with World Trade Organisation requirements [60].

Considering the influence exerted by advertising on young people’s food preferences, PICTs can implement recommendations to restrict the marketing of unhealthy products to children and adolescents [61]. PICTs can also ensure schools provide safe drinking water, and that school policies support physical activity opportunities [62] and improve the nutritional quality of food and drinks in school canteens [63]. Urban planning options may be explored which enable active transport and increase community recreational facilities and opportunities that are safe, accessible and acceptable for all genders. Prioritisation of the full array of options is required, a process which will be informed by the work of the WHO Commission on Ending Childhood Obesity.

Additional research on the similarities and differences between PICTs in the determinants of obesity and overweight is needed, in order to understand and account for the regional variation in adolescent prevalence. The inter-country variation may be indicative of different stages of the epidemiological transition; in islands where prevalence is lower, there may be a potential rise in obesity to come. For PICTs continuing to face the burden of infectious disease and under-nutrition, alongside increasing risks of overweight and obesity, an integrated approach to improve young people’s health and nutrition, focusing on healthy growth, is invaluable [64, 65].

To comprehensively and effectively tackle the NCD crisis, all sectors and agencies must be engaged. The Pacific NCD Road Map clearly describes the synergistic roles and responsibilities of different Ministries in tackling the NCD crisis, including the Prime Minister’s Office, Ministries of Trade, Finance, Agriculture, Fisheries, Customs and Excise, Education and Communications [66]. Furthermore, considering the influence that development organisations exert on the determinants of health, inter-agency coordination and harmonisation of such action is vital. The Pacific NCD Partnership, which brings together multiple agencies in the region, has recently been established to achieve this objective [67].

## Conclusion

Immediate action on obesity in the Pacific is imperative. Community based initiatives and population health approaches need to be sufficiently supported by national leadership, sustained funding and strengthened surveillance systems for monitoring the prevalence of obesity and its determinants, in addition to the inequalities within [63]. Both national and regional action are necessary to prevent NCDs from overwhelming health systems and jeopardising sustainable development in the islands. Such concerted efforts will be instrumental in preventing NCDs, including obesity and its consequences, and will enable PICTs to realise the vision of Healthy Islands [2].
